# Persistent functional impairment as an early indicator of cognitive decline and dementia in cognitively normal older adults

**DOI:** 10.1177/13872877251406661

**Published:** 2025-12-22

**Authors:** Maryam Ghahremani, Eric E. Smith, Zahinoor Ismail

**Affiliations:** 1 Psychiatry, Cumming School of Medicine, University of Calgary, Calgary, AB, Canada; 2 Hotchkiss Brain Institute, Cumming School of Medicine, University of Calgary, Calgary, AB, Canada; 3 Clinical Neurosciences, Cumming School of Medicine, University of Calgary, Calgary, AB, Canada; 4 Community Health Sciences, Cumming School of Medicine, University of Calgary, Calgary, AB, Canada; 5 O’Brien Institute for Public Health, Cumming School of Medicine, University of Calgary, Calgary, AB, Canada; 6 Clinical and Biomedical Sciences, Faculty of Health and Life Sciences, University of Exeter, Exeter, UK

**Keywords:** Alzheimer's disease, cognitive decline, dementia, dementia prognostication, functional impairment, symptom persistence

## Abstract

**Background:**

Functional independence is crucial for healthy aging, and its loss is a diagnostic criterion for dementia, including Alzheimer's disease. However, functional impairment (FI) can emerge before dementia diagnosis. Early and accurate characterization of FI may help identify individuals at elevated risk of cognitive decline and dementia.

**Objective:**

Exploring the utility of capturing persistent versus impersistent FI, to identify a higher-risk group for incident cognitive decline and dementia.

**Method:**

Data from 11,793 cognitively normal (CN) older adults from the National Alzheimer's Coordinating Center were analyzed. Exploratory factor analysis identified four Functional Activities Questionnaire items—preparing hot drinks, preparing balanced meals, shopping, and traveling—representing primarily functional abilities. An FI composite score was calculated as the sum of these items. Persistent FI was operationalized as FI present (composite score ≥ 2) at more than two-thirds of all visits prior to cognitive decline and dementia. Comparator groups were impersistent/transient FI and no FI. Time-dependent covariate Cox models compared incidence of cognitive decline and dementia across time-dependent FI groups, adjusted for demographics, *APOE* ε4 status, presence of neuropsychiatric symptoms, CDR sum of boxes, and informant characteristics (age, sex, relationship, cohabitation status).

**Results:**

The CN sample comprised 164 Persistent-FI (age = 75.7 ± 12.2; 59.1% female), 522 Transient-FI (age = 73.7 ± 9.5; 62.8% female), and 11,107 No-FI participants (age = 70.9 ± 8.9; 66.0% female). Persistent-FI was associated with a 2.12-fold greater incidence of cognitive decline and dementia versus No-FI (CI:1.80–2.51, p < 0.001). Transient-FI showed no significant difference (HR = 1.14, CI:0.97–1.33, p = 0.107).

**Conclusions:**

Operationalizing FI-related risk based on persistence is a promising approach to incorporation of FI into dementia prognostication.

## Introduction

With progression along the cognitive continuum from normal cognition to dementia, the ability to independently perform instrumental and basic activities of daily living (IADL and BADL) gradually diminishes. This loss of functional independence is a key criterion for the diagnosis of dementia.^1^ IADL encompass activities essential for independent living within the community, such as managing medications, preparing meals, shopping, managing personal finances, and traveling, all of which often require a certain level of cognitive processing. In contrast, BADL refer to fundamental skills necessary for managing basic physical needs, such as personal hygiene, eating, dressing, and toileting.^2^ Given their complexity and reliance on higher cognitive functions, IADL tend to be more susceptible to early brain pathological events compared to BADL.^3^ Consequently, impairments in IADL performance can manifest in advance of dementia, potentially serving as an early indicator of cognitive decline.

Along the cognitive continuum of dementia, mild cognitive impairment (MCI) was originally characterized as a stage with objective cognitive decline but maintenance of functional independence. However, a growing body of literature suggests that functional disabilities can be present in MCI^4–7^ and difficulty in performing ADL without losing functional independence is now part of the MCI diagnostic criteria of the National Institute on Aging and Alzheimer's Association (NIA-AA) research framework.^1^ Furthermore, longitudinal studies have demonstrated that difficulty in performing ADL in older adults with MCI could predict incident dementia.^8–11^ However, studies are limited on the association of functional impairment (FI) with incident dementia in those with objectively normal cognition,^3,12^ partly due to a lack of sensitive and accurate assessments that can detect early functional changes in this population. When accurately detected, FI presenting in advance of objective cognitive impairment could serve as an early marker of incident dementia in cognitively normal (CN) individuals. However, most of the existing FI assessment instruments lack the required sensitivity to detect subtle functional changes in the early stages of dementia. Importantly, existing instruments may fall short in appropriately accounting for the natural history of functional changes, to distinguish between transient non-pathological changes in function and persistent FI that could signal an underlying neurodegenerative process. Transient FI may reflect short-term disruptions unrelated to underlying neurodegenerative processes. Such changes can result from acute medical conditions (e.g., infections, pain exacerbations), medication effects, sleep disturbances, or short-term psychological stressors. In these instances, the impairment in functional abilities may represent a temporary reduction in capacity or motivation rather than an early sign of progressive decline due to neurodegeneration. Similarly, evidence has shown that persistent new-onset behavioral changes are more likely to be linked to an underlying neurodegenerative disease process than conventionally measured behavioral symptoms.^13–15^ This same reasoning may apply to FI. Recognizing such limitations in traditional assessments of function, our study explored a similar paradigm that incorporated the natural history into classification of function. We investigated the association of FI, both persistent and transient, with incident cognitive decline and dementia in CN older adults to better understand how different patterns of FI may signal risk for future cognitive decline and dementia. The identification of persistent FI as an early disease marker could provide a valuable tool for early intervention and improved prognostication, allowing for the identification of CN individuals at higher risk for developing dementia.

## Methods

### Study population

Data were obtained from the National Alzheimer's Coordinating Centre (https://naccdata.org), with a March 2024 data freeze. NACC was established by the National Institute on Aging (NIA) and consists of multiple NIA-funded AD Research Centers (ADRCs) recruiting and collecting data from individuals with cognitive function ranging from normal cognition to dementia. The NACC Uniform Data Set (UDS) is a large prospective and longitudinal clinical evaluation that includes demographic and standardized clinical data collected approximately annually. All contributing ADRCs were required to administer standardized forms, obtain informed consent from participants and their informants, and IRB approvals prior to submitting data to NACC. Detailed information on the cohort is described elsewhere.^16–18^

### Participant selection

**Figure 1.**
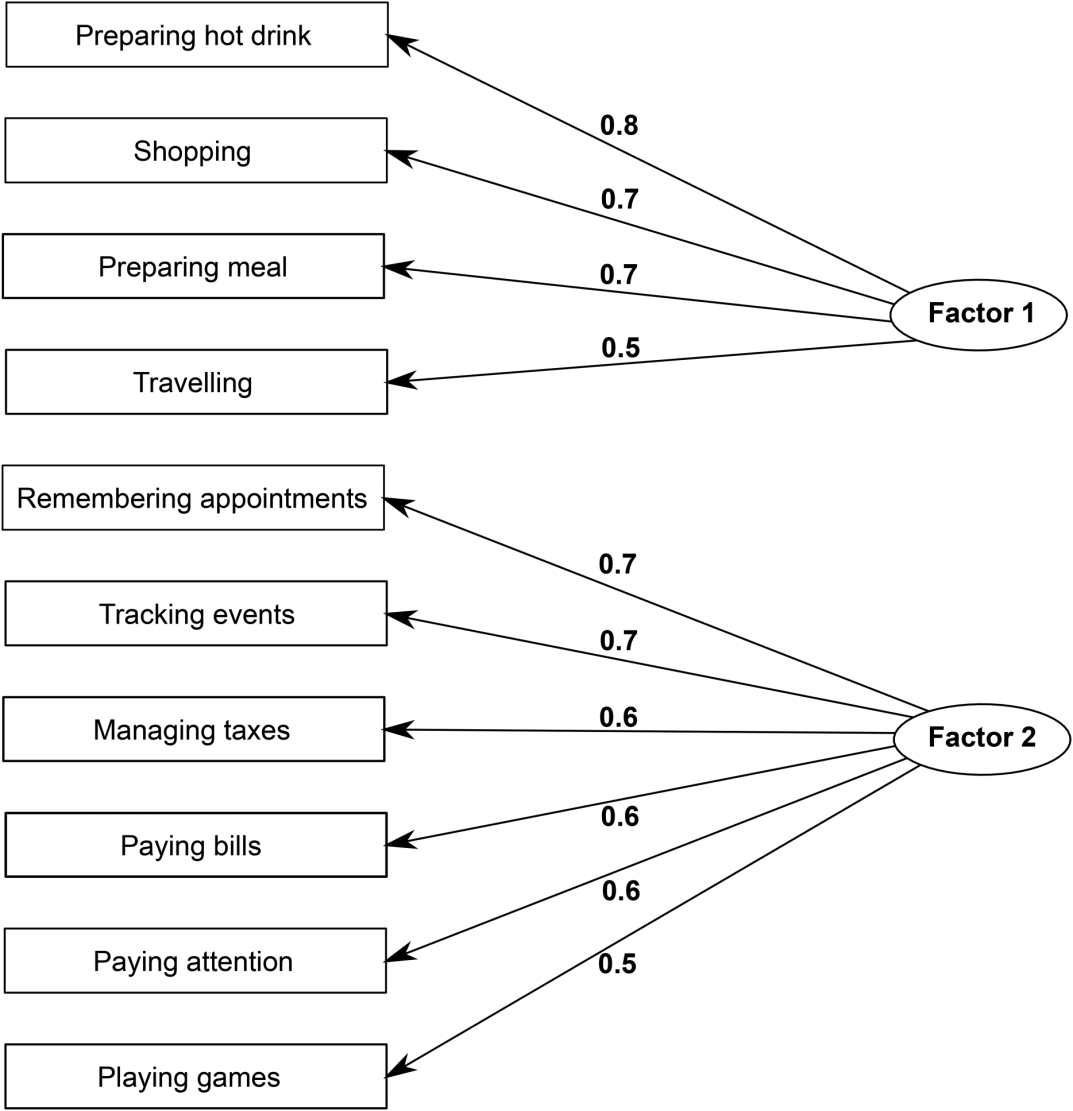
**Exploratory factor analysis of the Functional Activities Questionnaire (FAQ) items.** The ten FAQ items were analyzed and loaded onto two distinct factors. Factor 1 is interpreted as representing functional abilities, while Factor 2 representing cognitive abilities. Numbers on the arrow lines represent factor loadings, which indicate the strength of the relationship between each FAQ item and each of the extracted factors.

Participants required complete Functional Activities Questionnaire (FAQ) data to determine FI status. FAQ is an informant-reported screening tool that assesses the level of dependence in performing ten IADL: paying bills, managing taxes, shopping alone, playing games, preparing a hot drink, preparing a balanced meal, tracking current events, traveling outside the neighborhood, paying attention, and remembering appointments and medications, scored from 0 (normal) to 3 (dependent).^19^ Acknowledging the overlap between cognition and function, we aimed to empirically identify FAQ items that best reflect day-to-day functional ability, as distinct from tasks that primarily assess memory or other cognitive domains. While all FAQ items involve some degree of cognitive processing, certain items, such as remembering appointments or managing finances, more directly index cognitive dysfunction and may be confounded with early cognitive decline. To reduce this overlap and better isolate functional ability, we conducted an exploratory factor analysis (EFA) on FAQ domain scores, averaged across all study visits prior to cognitive decline and dementia for each participant. A scree plot and eigenvalue inspection supported a two-factors solution as the most appropriate for the data. Based on the EFA results, we identified four items—preparing a hot drink, preparing a balanced meal, shopping alone, and traveling outside the neighborhood—that loaded onto a factor representing instrumental functional ability (Figure 1). While these tasks still engage certain level of cognitive processes such as executive function and orientation, they represent IADL that more closely reflect practical functional independence. The four FAQ items identified by the EFA—preparing a hot drink, preparing a balanced meal, shopping alone, and traveling outside the neighborhood—were used to define an FI composite score calculated as the sum of individual item scores, which was used in the subsequent analyses to assess FI. However, to further minimize the potential influence of cognitive decline on assessment of function, analyses adjusted for cognitive status using a modified Clinical Dementia Rating (CDR) scale measure, referred to as CDR-MOJ. This score included only the CDR memory, orientation, and judgement/problem solving domains, cognitive domains likely involved in IADL performance. CDR functional domains—community affairs, home and hobbies, and personal care—were excluded to avoid redundancy with functional impairment as measured by the FAQ.

Persistent-FI was operationalized as FI composite score ≥ 2^20^ at more than two-thirds of all study visits (TTV) prior to cognitive decline and dementia, based on a validated approach previously applied in the context of using neuropsychiatric symptoms (NPS) to operationalize mild behavioral impairment (MBI).^21^ The two comparator groups either had transient FI not meeting TTV criteria (Transient-FI) or an FI score of zero (No-FI) in advance of cognitive decline and dementia. In all FI groups, the NACC clinical cognitive diagnosis was used to ensure only CN participants at baseline were included, based on a global CDR scale (CDR^®^ Dementia Staging Instrument) score of 0 and/or neuropsychological testing within normal range. Participants below the age of 50 and those with movement disorders (Parkinson's and Huntington's diseases) or neurodevelopmental disorders (Down's syndrome) were excluded. Participants were required to have at least one follow-up visit and available data on covariates of interest for longitudinal modeling. These covariates included age, sex, education years, *APOE* ε4 status, NPS profile, CDR-MOJ scores, and informant characteristics. *APOE* ε4 status was categorized as carrier (one or two ε4 copies) and noncarrier. NPS profile was defined as the sum of the following ten items of the Neuropsychiatric Inventory Questionnaire (NPI-Q) as captured in MBI^22,23^: apathy/indifference, depression/dysphoria, anxiety, elation/euphoria, agitation/aggression, irritability/liability, aberrant motor behavior, disinhibition, delusions, and hallucinations. An NPS total score > 0 at each visit was considered NPS+, while a score of zero was considered NPS-. The informant characteristics included age, sex, relationship to the participant, and cohabitation status. The informant relationships were classified into three categories: spouse, child, and other. The “other” category encompassed less common relationships, such as siblings, extended family members, friends, paid caregivers, and healthcare providers, lumped together due to small sample size per category. For time-independent model covariates (age, sex, education years, *APOE* ε4 status, as well as informant's age, sex, relationship to the participant, and cohabitation status) available data were required only at baseline, whereas for time-dependent model covariates (FI status, NPS profile, and CDR-MOJ score) data were required across all study visits preceding cognitive decline and dementia.

**Figure 2.**
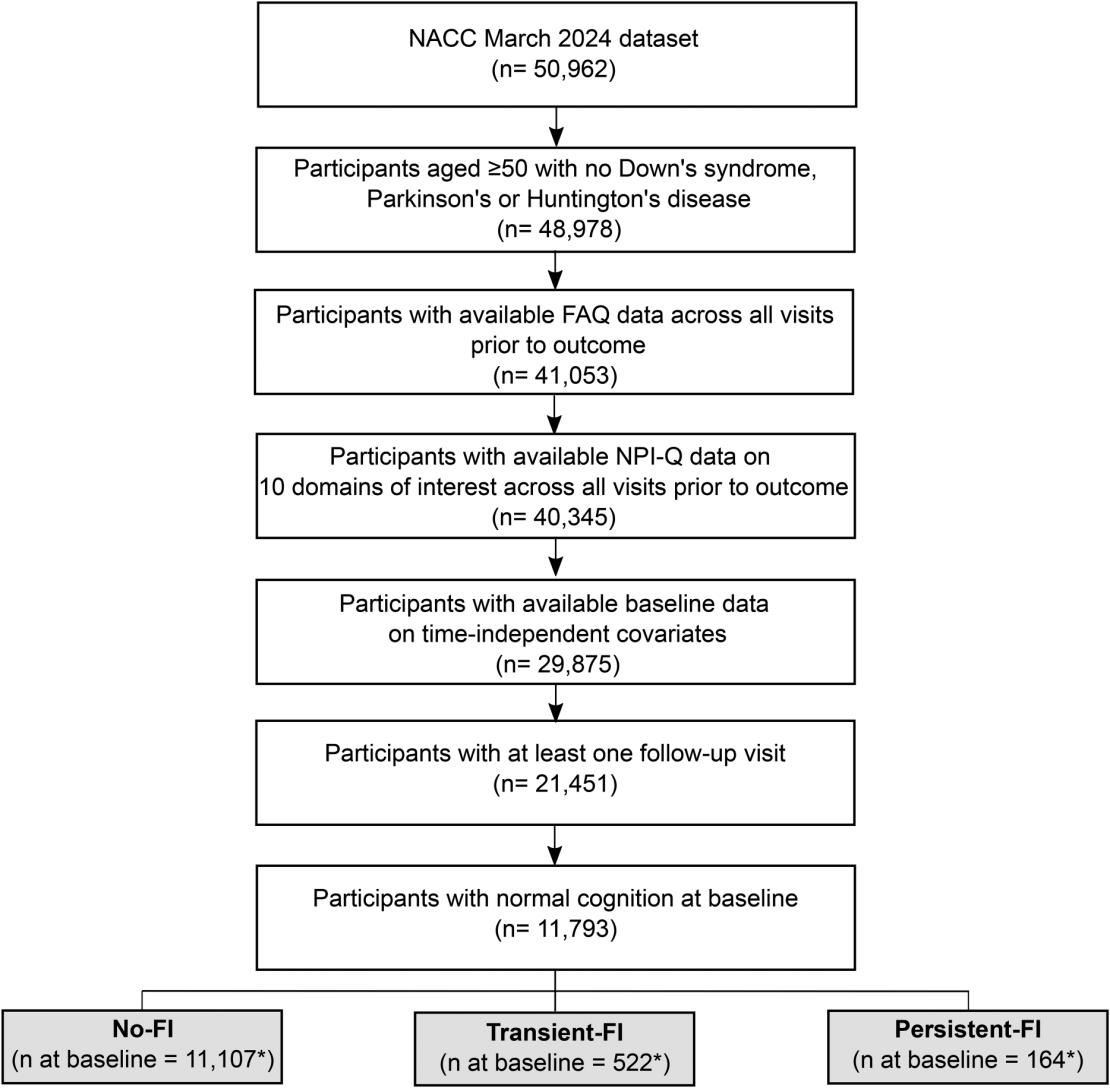
Flowchart: participant selection process. FAQ: Functional Activities Questionnaire; FI: functional impairment; NPI-Q: Neuropsychiatric Inventory Questionnaire. *FI status is a time-dependent covariate and therefore the sample size per FI group may change across study visits. The sample size shown in the flowchart reflects the baseline sample only.

The outcome measure, diagnosis of MCI and dementia, was based on the NACC clinical cognitive diagnosis, made by either a consensus panel or a single physician. According to the D1 clinical diagnosis form in NACC, diagnosis of MCI requires a subjective cognitive complaint and objective impairment on a cognitive test in one or more cognitive domains, with largely preserved independence in functional abilities. Dementia diagnosis requires cognitive or behavioral symptoms that interfere with the ability to function in IADL, are not explained by major psychiatric disorders, and include cognitive impairments detected through history taking and cognitive assessment. Figure 2 illustrates the participant selection process.

### Statistical analysis

Baseline age, sex, education years, *APOE* ε4 status, NPS profile, CDR-MOJ scores, as well as informant characteristics were compared across FI groups at baseline to identify significant differences, using t-tests for continuous variables and Chi-squared tests for categorical variables. It is important to note that the values for time-dependent covariates (FI status, NPS profile, and CDR-MOJ scores) can change over study visits, reflecting their dynamic nature throughout the study period.

Time-dependent covariate Cox models were utilized to determine hazard ratios (HRs) for progression from normal cognition to MCI or dementia. The time-dependent model covariates included FI status, NPS profile, and CDR-MOJ score, which were updated at each study visit prior to the outcome, allowing for a more robust and accurate modeling of the risk associated with the outcome. Age, sex, education years, *APOE* ε4 status, and informant characteristics were considered as time-independent covariates, which were only captured at baseline. All HRs were accompanied by 95% confidence intervals (CI) and p-values.

The interaction of FI status with sex, *APOE* ε4 status, NPS profile, CDR-MOJ score, and informant characteristics were explored to identify potential effect modification across strata of each covariate. Multiplicative interaction tests were implemented to assess between-strata differences in the observed estimates.

All statistical analyses were performed in RStudio version Prairie Trillium (2022.02.0) (R version 4.2.2), using the *psych* package for factor analysis, *survival* package for Cox models, and *ggplot2* and *survminer* for forest plots.

## Results

The final sample of 11,793 CN participants comprised 11,107 participants with no FI prior to the onset of cognitive decline and dementia (mean age 70.9 ± 8.9; 66.0% female), 522 with Transient-FI (mean age 73.7 ± 9.5; 62.8% female), and 164 with Persistent-FI (mean age 75.7 ± 12.2; 59.1% female) at baseline. Both Transient-FI and Persistent-FI groups differed significantly from the No-FI group in terms of age (p < 0.001), education years (Transient-FI: p = 0.012; Persistent-FI: p < 0.001), NPS profile (p < 0.001), and mean CDR-MOJ scores (p < 0.001). The No-FI group was younger and more educated. NPS were more prevalent in both Transient-FI and Persistent-FI, compared with No-FI, with the highest prevalence in Persistent-FI. Mean CDR-MOJ score was higher in both Transient-FI and Persistent-FI in comparison to No-FI, with the highest score in Persistent-FI. Participant sex or *APOE* ε4 status did not differ significantly across FI groups. In terms of informant characteristics, the Transient-FI group had younger informants (p = 0.043), and a higher proportion of female informants (p = 0.002) compared to the No-FI group, though these differences were not significant in the Persistent-FI group. Both Transient-FI and Persistent-FI groups had a higher proportion of child informants, and a lower proportion of spouse informants compared to the No-FI group (p < 0.001). There were no significant differences in informant cohabitation status across FI groups (Table 1).

**Table 1.** Sample demographics: baseline demographic characteristics of cognitively normal participants across functional impairment groups. Between group differences were assessed using t-tests for continuous variables and Chi-squared tests for categorical variables.

	No-FI (N = 11,107)	Transient-FI (N = 522)	Persistent-FI (N = 164)	Transient vs. No-FI p-value	Persistent vs. No-FI p-value
Age					
Mean (SD)	70.9 (8.9)	73.7 (9.5)	75.7 (12.2)	**<0.001**	**<0.001**
Sex					
Female	7335 (66.0%)	328 (62.8%)	97 (59.1%)	0.144	0.077
Male	3772 (34.0%)	194 (37.2%)	67 (40.9%)		
Years of education					
Mean (SD)	16.0 (2.8)	15.7 (2.8)	14.4 (3.7)	**0.012**	**<0.001**
*APOE* ε4 status					
Noncarrier	7587 (68.3%)	358 (68.6%)	118 (72.0%)	0.934	0.362
Carrier	3520 (31.7%)	164 (31.4%)	46 (28.0%)		
NPS group					
NPS-	8528 (76.8%)	213 (40.8%)	49 (29.9%)	**<0.001**	**<0.001**
NPS+	2579 (23.2%)	309 (59.2%)	115 (70.1%)		
CDR-MOJ					
Mean (SD)	0.0775 (0.238)	0.446 (0.601)	0.814 (0.900)	**<0.001**	**<0.001**
Informant					
Age					
Mean (SD)	63.0 (13.7)	61.8 (14.0)	62.2 (13.0)	**0.043**	0.425
Sex					
Female	6861 (61.8%)	358 (68.6%)	111 (67.7%)	**0.002**	0.143
Male	4246 (38.2%)	164 (31.4%)	53 (32.3%)		
Relationship					
Spouse	5678 (51.1%)	249 (47.7%)	73 (44.5%)	**<0.001**	**<0.001**
Child	2431 (21.9%)	163 (31.2%)	58 (35.4%)		
Other	2998 (27.0%)	110 (21.1%)	33 (20.1%)		
Cohabitation					
No	4971 (44.8%)	240 (46.0%)	69 (42.1%)	0.615	0.544
Yes	6136 (55.2%)	282 (54.0%)	95 (57.9%)		

CDR-MOJ: clinical dementia rating scale sum of memory, orientation, and judgement domains; FI: functional impairment; NPS: neuropsychiatric symptoms; SD: standard deviation.

Bold p-values indicate statistical significance (p < 0.05).

**Figure 3.**
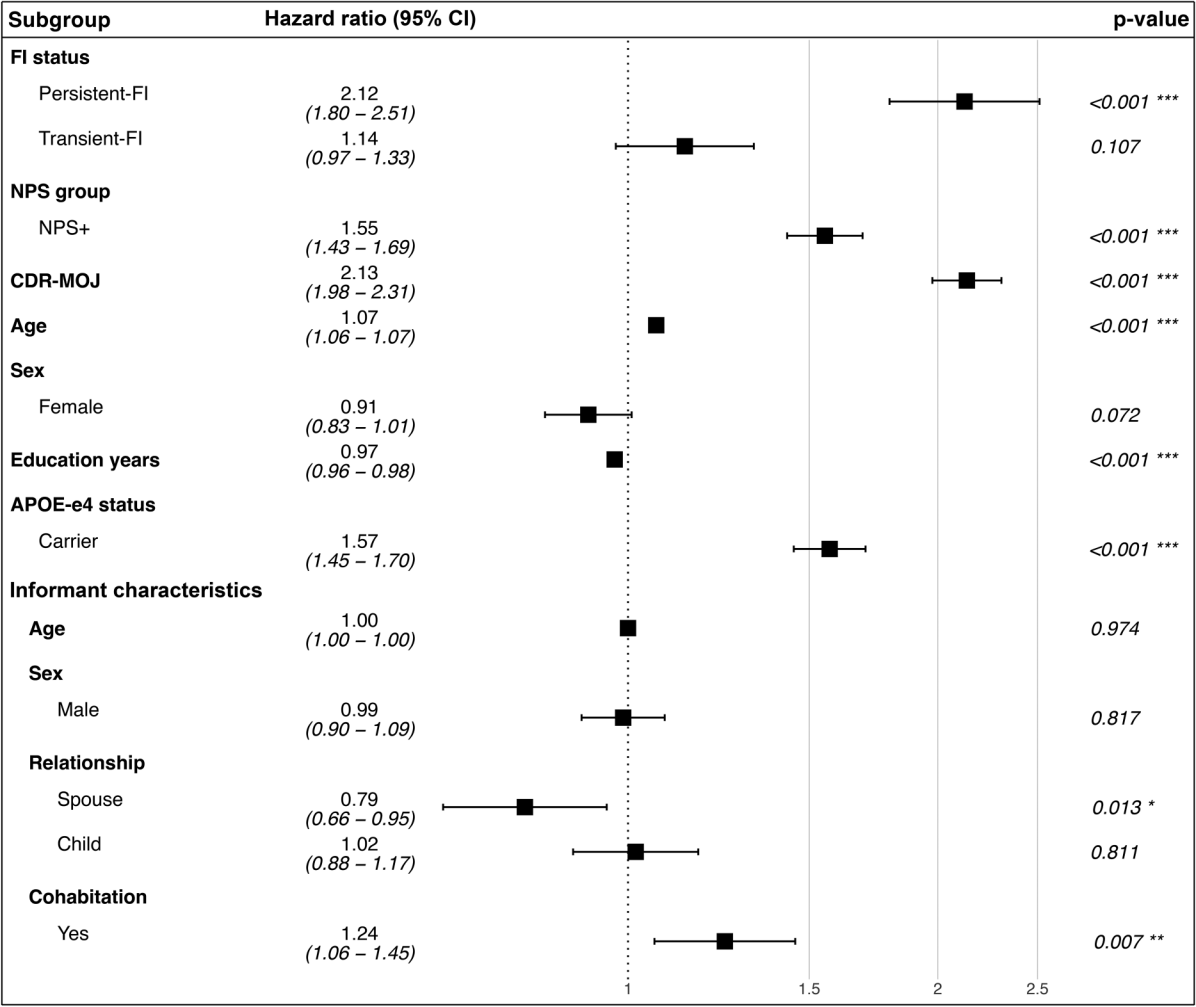
Forest plot: hazard ratios for incident cognitive decline and dementia across functional impairment groups, adjusted for CDR sum of memory, orientation, and judgement domains and neuropsychiatric symptoms profile as time-dependent covariates, and age, sex, education years, *APOE* ε4 status, and informant characteristics (age, sex, relationship, cohabitation) as time-independent covariates. CDR-MOJ: clinical dementia rating scale sum of memory, orientation, and judgement domains; CI: confidence interval; FI: functional impairment; NPS: neuropsychiatric symptoms.

Compared with the No-FI group, the Persistent-FI group had a 2.12-fold greater adjusted hazard for MCI or dementia (CI:1.80–2.51, p < 0.001). In contrast, the Transient-FI group had a HR of 1.14 (CI:0.97–1.33, p = 0.107), which did not significantly differ from No-FI. NPS at baseline were associated with a 1.55-fold greater incidence of MCI or dementia than no NPS (CI:1.43–1.69, p < 0.001), and higher baseline CDR-MOJ scores were associated with a 2.13-fold greater incidence than lower scores (CI:1.98–2.31, p < 0.001). Among model covariates related to informant characteristics, having a spouse informant was associated with a lower incidence of MCI or dementia (HR = 0.79, CI:0.66–0.95, p = 0.013), while cohabiting with the participant was associated with a higher incidence (HR = 1.24, CI:1.06–1.45, p = 0.007) (Figure 3). No significant interaction of FI group was found with participant sex, *APOE* ε4 status, or any informant characteristics. However, the association between FI status and incidence of MCI or dementia differed between the strata of NPS profile, such that the relative impact of FI was lower within the NPS + group than in the NPS- group. These results were significant for both Transient-FI (Transient-FI NPS + versus Transient-FI NPS-: HR = 0.69, CI:0.51–0.94, p = 0.02) and Persistent-FI groups (Persistent-FI NPS + versus Persistent-FI NPS-: HR = 0.71, CI:0.52–0.98, p = 0.04). Similar interaction effects were observed between FI status and CDR-MOJ score, such that the relative impact of FI was lower among participants with above mean CDR-MOJ scores than those scores below the mean. These results were significant for both Transient-FI (Transient-FI higher versus lower CDR-MOJ scores: HR = 0.49, CI:0.39–0.60, p < 0.001) and Persistent-FI (Persistent-FI higher versus lower CDR-MOJ scores: HR = 0.44, CI:0.37–0.53, p < 0.001).

## Discussion

In this longitudinal study of 11,793 CN participants, individuals with persistent FI had a notably greater incidence of MCI or dementia compared to those with transient or no FI. Our study demonstrated that operationalizing function-related risk based on FI persistence significantly improved prognostication for cognitive decline and dementia, enabling early identification of high-risk individuals even in the absence of measurable cognitive decline. Our findings hold significant implications for both clinical trials and clinical practice.

IADL impairment has been widely reported in MCI. Systematic reviews have revealed that individuals with MCI exhibit greater IADL difficulties compared to CN, particularly in managing finances, shopping, driving, and maintaining appointments.^4,6^ IADL impairments are also associated with greater amyloid-β (Aβ) burden in MCI, compared to CN individuals.^24,25^ Furthermore, in MCI, IADL impairment was associated with a higher likelihood of progression to dementia than those without impairment, indicating greater specificity for predicting incident dementia when combining MCI with IADL impairment.^8,9,^^26–28^ A population study of dementia-free older adults identified ADL disability (encompassing BADL and IADL) as a significant predictor of incident dementia after adjusting for baseline cognition.^11^ These results may not be surprising, since IADL are complex by definition and therefore decline in IADL performance can be driven by emerging cognitive decline and early pathological changes in the brain.^29^ However, few studies have investigated IADL disability emerging in advance of objective cognitive decline. Focusing on IADL, rather than BADL, in cognitively unimpaired populations is particularly important because these IADL rely on higher-order cognitive and executive functions, making them more sensitive indicators of early neurodegenerative disease than BADL. This emphasis on IADL enables the identification of individuals at risk for cognitive decline even before overt cognitive impairment is detectable.

Given that IADL are particularly sensitive to early higher-order cognitive changes, evidence of IADL impairment has been reported earlier along the cognitive continuum of dementia, including in older adults with normal cognition or subjective cognitive decline (SCD). In a study assessing the relationship between SCD and IADL performance, participants with higher Aβ burden demonstrated greater impairment in performing IADL.^30^ Similar findings have been observed in CN older adults.^31^ These findings are consistent with the literature that links Aβ plaques to lower IADL abilities in preclinical and prodromal Alzheimer's disease.^25,32^ Few studies have reported on the association of FI with Aβ burden in CN older adults. A cross-sectional study using the Financial Capacity Instrument Short Form (FCI-SF) demonstrated that in CN participants, greater Aβ burden was associated with greater difficulties in completing financial tasks.^33^ In a more recent cross-sectional study of CN older adults, IADL difficulties were associated with mildly higher Aβ burden and lower cognitive performance at baseline.^34^ One longitudinal study of cognitively unimpaired older adults reported that handling finances and medications were sensitive to cognitive decline in those who later developed the Aβ neuropathology.^12^ Another longitudinal study of dementia-free older adults (59% CN and 41% with MCI) revealed a trend towards deteriorating IADL performance among Aβ+ participants compared to Aβ-.^35^ These results indicate that even in the preclinical stages of dementia, there is a link between IADL and Aβ burden.

Our findings on the association of persistent FI with incident cognitive decline and dementia in CN older adults add to a scarce body of literature, suggesting that accurately captured FI could represent one of the earliest manifestations of dementia-related pathology. Compared to previous studies, the present study took a unique perspective in capturing FI by considering the persistence of FI across multiple study visits prior to cognitive decline and dementia, to differentiate from transient impairments possibly not linked to underlying disease. A limitation of existing FI assessment scales is the lack of a well-defined temporal reference point for symptoms. For instance, scales such as the Bayer ADL,^36^ Bristol ADL,^37^ Lawton-Brody ADL,^38^ and Amsterdam IADL Questionnaire^39^ do not specify a timeframe for assessing FI, while the disability assessment for dementia scale^40^ only references a two-week timeframe. While the original FAQ scale^19^ does not specify a timeframe, the FAQ included in NACC B7 form has been modified to add a four-week timeframe. Assessing FI at a single time point or within a short timeframe makes it challenging to discern whether the observed impairment reflects temporary change due to an external stressor or secondary cause or represents a decline in performance associated with a neurodegenerative disease process. By capturing the persistence of symptoms over at least two-thirds of annual study visits in advance of cognitive decline,^21^ our study was able to identify a strong association between FI and incident cognitive decline and dementia, in the absence of objective cognitive decline. In contrast, Transient FI not meeting the persistence criterion failed to capture any significant risk, likely due to the noise associated with temporary functional impairments caused by multiple etiologies. Importantly, translating the observed association between Persistent-FI and greater risk of cognitive decline and dementia into practice requires careful consideration of how persistence is defined and measured. In real-world settings with irregular visit frequency, persistence could be operationalized as functional difficulties reported across at least two consecutive assessments separated by several months. Additionally, short, targeted IADL questionnaires that are designed to inherently capture the natural history of symptoms (e.g., symptom presence for at least six months), could facilitate practical measurement even in resource-limited environments. Such brief tools could be administered in person, by phone, or via digital platforms, enabling clinicians to efficiently identify individuals who may benefit from closer cognitive monitoring or early intervention. Nonetheless, this temporal dimension of FI in our study has important translational implications, as findings could inform real-world applications to identify at-risk individuals and guide early interventions. Behavioral nudging tools could leverage these insights to provide support for at-risk individuals, repeatedly targeting the specific activities most affected over time. Similarly, smart home technologies could monitor longitudinal patterns in daily functioning, detecting sustained decline rather than transient lapses. Community health interventions, including occupational therapy or structured support programs, could prioritize individuals with FI persisting over time, enabling timely, targeted strategies to maintain independence and potentially slow cognitive deterioration.

Among the ten items of the FAQ, our EFA identified four items of preparing a hot drink, preparing a balanced meal, shopping alone, and traveling outside the neighborhood as reflecting instrumental functional ability. Previous studies have indicated that FAQ items of “remembering appointments” and “assembling tax records” best distinguished between CN and MCI individuals.^41,42^ Items like “paying attention to and understanding a TV program”, “paying bills/balancing checkbook”, and “heating water and turning off the stove after use” best predicted progression from CN to MCI.^41^ While all these identified items involve certain degree of cognitive processing (e.g., attention, executive function, and memory), some items more directly assess cognitive symptoms rather than functional capacity. For example, “remembering appointments” and “paying attention to and understanding a TV program” primarily reflect deficits in memory and attention, and their endorsement may indicate early cognitive decline rather than reduced functional independence. As such, it is no surprise that a lower score on these FAQ items distinguishes between CN and MCI, essentially capturing the decline in cognition as one progresses to MCI. Conversely, FAQ items such as preparing a drink or a meal, shopping, and traveling, while still dependent on cognitive support, are more directly related to functional independence and the ability to carry out IADL. Cognitive abilities are certainly involved in completing all FAQ tasks to some extent; the distinction lies in whether the task primarily assesses functional or cognitive ability. Given this distinction, our study aimed to narrow the focus on the FAQ items less reliant on cognitive function, to investigate the specific role of functional impairment as a potential early marker of cognitive decline and dementia. To further isolate functional ability, models were adjusted for CDR memory, orientation, and judgment/problem-solving scores, minimizing the influence of cognitive decline on observed associations.

In addition to FI status, informant characteristics in our model were independently associated with incident cognitive decline and dementia. Having a spouse as the informant, compared to a child, was linked to a lower incidence of cognitive decline and dementia, whereas cohabitation with the participant was associated with a higher incidence. It is important to note that a spouse can also be a cohabitant; our model separated these factors to distinguish the influence of relationship type from living situation. These findings likely reflect differences in how informants observe and report functional changes, suggesting that cohabitation may provide a more precise view of functional impairment. Spouses, who may or may not cohabit, are often highly familiar with the participant's baseline functioning. While this long-term familiarity might allow them to notice gradual changes, spouses may also tend to normalize small functional difficulties or actively compensate for them, which can mask early functional difficulties until deficits become more pronounced. However, cohabitation with the participant allows for more direct observation of daily fluctuations and subtle lapses in functional abilities, leading to more sensitive identification of early deficits. Cohabitation may also indicate greater baseline care needs, capturing participants already on a faster trajectory of decline. Collectively, these findings highlight that informant relationship and living situation influence how functional decline is detected and reported. Nonetheless, after adjusting for these informant-related factors, the strong association between persistent FI and incident cognitive decline and dementia remained robust, reinforcing the independent predictive value of natural history of FI.

In our study, the crude rate of progression to cognitive decline and dementia was expectedly higher in NPS + than NPS- (HR = 1.55), and in those with CDR-MOJ scores above the mean (HR = 2.13) (Figure 3). However, compared to no FI, the relative effect of persistent FI on the rate of incident cognitive decline and dementia was lower in NPS+ than in NPS- and in those with CDR-MOJ scores above the mean than below. These findings suggest that within our sample, persistent FI emerged as the primary driving factor to the risk of incident cognitive decline and dementia, outweighing the relative contributions of other model covariates.

It is important to note that NPS in our analysis were assessed at a single timepoint, potentially resulting in a less precise measure of dementia-related NPS. Future studies should incorporate temporal NPS measures into their models, utilizing assessment scales that account for symptom natural history, such as the MBI checklist (MBI-C).^43,44^ The MBI core criteria of later-life emergence and persistence of symptoms have demonstrated utility in identifying persons with NPS with poorer cognition^45^ and at highest risk for incident cognitive decline and dementia.^46,47^ In MCI, for example, those with persistent NPS meeting MBI criteria were more likely to progress to dementia and less likely to revert to normal cognition than those with transient NPS.^23^ Similarly, persons with later-life emergent and persistent NPS demonstrated neurodegenerative disease-related changes on imaging,^48–50^ and a greater burden of AD neuropathology compared to those with impersistent/transient NPS.^14,15,51^ Indeed, the approach used to successfully validate MBI as a pre-dementia risk marker informed our study of function. Thus, the successful application of this strategy to FI highlights the need for more specific risk measures to enhance dementia prognostication. Hence, the approach used to demonstrate that appropriately ascertained behavior, and now function, improve dementia prognostication could be applicable to other markers, warranting further research.

### Limitations

One limitation of the present study is the use of FAQ to assess FI in a CN sample. FAQ was designed for dementia populations, limiting its sensitivity to subtle FI in preclinical and prodromal dementia.^4^ Although FAQ is informant-reported due to cognitive impairments in dementia, self-reported FI scales could be considered for CN samples. Informant-reported FI may be influenced by characteristics of the informant, including age, sex, relationship type, and cohabitation status, which may contribute to measurement bias in informant-based assessments. However, both self-reported and informant-reported IADL are subject to biases such as mood and personality and therefore, performance-based measures may provide more reliable report of symptoms,^52,53^ although are not scalable, and cannot be administered remotely. Moreover, FAQ is limited in its coverage of activities to reflect modern IADL such as technology use,^54^ which are covered in other scales.^39^ Another limitation is the challenge of distinguishing IADL difficulties secondary to underlying neurodegeneration from those arising from physical, sensory, or other health-related conditions. To address this ambiguity, we conducted additional analyses that included a covariate capturing physical and sensory limitations (hearing, vision, gait, and slowness). Results were nearly identical to the original models, with Persistent-FI remaining strongly associated with incident cognitive decline and dementia, while Transient-FI was not significantly associated (Supplemental Figure 1). These unchanged results support the robustness of our findings and suggest that the observed associations primarily reflect functional deficits related to underlying neurodegenerative processes rather than physical or sensory limitations. Nevertheless, we acknowledge that other comorbid medical conditions, such as arthritis, cardiovascular disease, or chronic pain, may also contribute to functional limitations independent of cognition. While these conditions were not directly modeled in the present study, future research incorporating comprehensive comorbidity and physical health measures would help clarify the relative contributions of such non-cognitive factors to early functional decline. Additionally, NACC participant demographics may limit the generalizability of our findings. Overall, compared to black and other racialized participants, white participants are more likely to live near academic centers, have greater trust and engagement with healthcare systems, and more flexibility to attend weekday study visits, which can facilitate enrollment and introduce selection bias. The overrepresentation of highly educated white participants in NACC^55^ may not reflect the entire spectrum of at-risk populations. In this context, it is critical to acknowledge the need for culturally sensitive validation of FI measures across diverse populations, as differences in daily routines, household roles, and cultural expectations can influence both functional performance and informant reporting. Future iterations of the NACC UDS aiming to improve representativeness will hopefully mitigate this bias and allow for studies that better account for racial differences in assessment of function.

Despite these limitations, findings highlight the importance of appropriately ascertained FI early in the disease course as a potential marker for dementia, as well as the translational relevance of persistent FI. In clinical practice, detecting persistent FI could help identify CN individuals at elevated risk for cognitive decline. In community settings, identifying persistent FI could inform targeted interventions to maintain independence and support functional abilities. Future research should prioritize the validation of established instruments and the development of novel scales tailored to assess FI in preclinical dementia, incorporating symptom persistence to enhance specificity in capturing FI related to the neurodegenerative processes of dementia.

### Conclusion

The present study provides strong evidence that operationalizing FI-related risk based on the persistence of FI improves the prognostication of cognitive decline and dementia, facilitating the identification of high-risk individuals before the onset of objective cognitive impairments. These findings highlight the importance of integrating IADL assessment into early dementia detection strategies, along with cognition and behavior. Given IADL sensitivity to early higher-order cognitive changes, this work emphasizes the need for further research to refine IADL assessment instruments in the preclinical stage. Detecting IADL impairments in CN older adults points to the potential value of functional assessments in preclinical AD prevention trials, which, like MBI, can be completed remotely by self or informant, enhancing scalability and reducing costs.

## Supplemental Material

sj-docx-1-alz-10.1177_13872877251406661 - Supplemental material for Persistent functional impairment as an early indicator of cognitive decline and dementia in cognitively normal older adults Supplemental material, sj-docx-1-alz-10.1177_13872877251406661 for Persistent functional impairment as an early indicator of cognitive decline and dementia in cognitively normal older adults by Maryam Ghahremani, Eric E. Smith and Zahinoor Ismail in Journal of Alzheimer's Disease
