# Supplementary material for: Persistent functional impairment as an early indicator of cognitive decline and dementia in cognitively normal older adults
Source: J Alzheimers Dis. 2025 Dec 22;109(3):1415–27. doi: 10.1177/13872877251406661 (PMC12855616; doi:10.1177/13872877251406661)
Supplement: sj-docx-1-alz-10.1177_13872877251406661 - Supplemental material for Persistent functional impairment as an early indicator of cognitive decline and dementia in cognitively normal older adults [file sj-docx-1-alz-10.1177_13872877251406661.docx]

**Supplemental Material**

**Persistent functional impairment as an early indicator of cognitive decline and dementia in cognitively normal older adults**

**Supplemental Figure 1. Forest plot:** hazard ratios for incident cognitive decline and dementia across functional impairments groups, adjusted for age, sex, education years, *APOE* ε4, CDR sum of memory, orientation, and judgement domains, neuropsychiatric symptoms profile, informant characteristics (age, sex, relationship, cohabitation) and an additional binary covariate capturing physical and sensory limitations (hearing, vision, gait, and slowness).

CDR-MOJ: clinical dementia rating scale sum of memory, orientation, and judgement domains; CI: confidence interval; FI: functional impairment; NPS: neuropsychiatric symptoms.
